# Entirely Bioderived Intumescent Flame-Retardant Coating for Polyamide Textiles

**DOI:** 10.1021/acspolymersau.6c00154

**Published:** 2026-08-26

**Authors:** Margaret J. Karim, Sarah G. Fisher, Kristin L. Smith, Dallin L. Smith, Jaime C. Grunlan

**Affiliations:** † Department of Chemistry, Texas A&M University, College Station, Texas 77843, United States; ‡ Department of Biochemistry and Biophysics, Texas A&M University, College Station, Texas 77843, United States; § Department of Materials Science and Engineering, Texas A&M University, College Station, Texas 77843, United States; ∥ Department of Mechanical Engineering, Texas A&M University, College Station, Texas 77843, United States

**Keywords:** nylon, self-extinguishing, polyelectrolytes, chitosan, tannic acid, guanidine phosphate

## Abstract

Polyamide 6,6 (PA6,6) is one of the most widely used synthetic textile fibers for consumer and industrial applications, but its inherent flammability and melt-dripping behavior present significant fire hazards. A fully bioderived intumescent flame retardant for PA6,6 textiles was developed using tannic acid (TA), chitosan (CS), and guanidine phosphate (GP). The coating was designed as an effective flame-retardant formulation that conformally coats PA6,6 fibers to minimally impact mechanical properties. Additionally, all materials used were derived from biological feedstocks to minimize the use of petroleum-derived materials. All coated PA6,6 samples eliminated melt dripping during vertical flame testing and achieved self-extinguishing behavior. The limiting oxygen index was increased from 23.5% to 27%, and the peak heat release rate measured by cone calorimetry was reduced by 56%. Overall, this work demonstrates an effective and scalable, bioderived, intumescent coating capable of significantly improving the fire safety of PA6,6 textiles.

## Graphical abstract

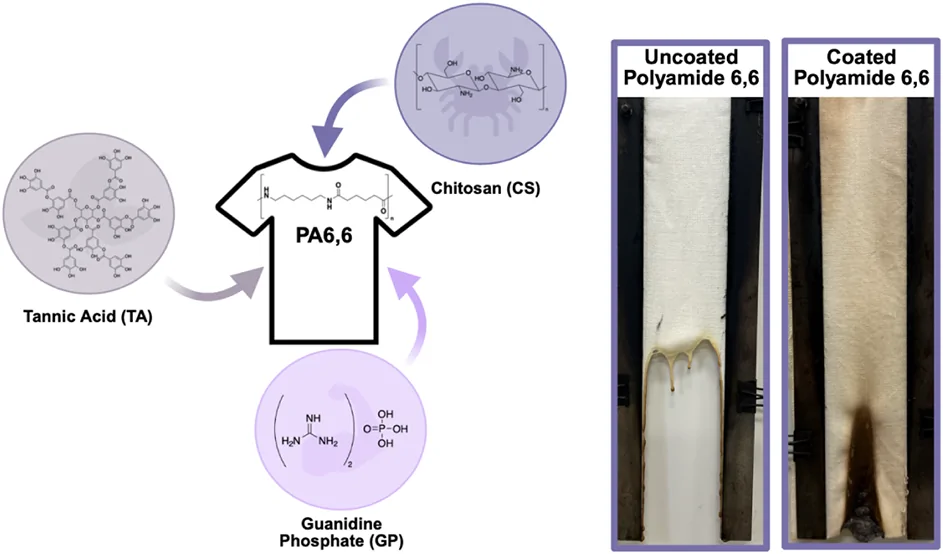

## 1 Introduction

Synthetic polyamides (PAs), often generally referred to as “nylon”, are known for their unique combination of high tensile strength, toughness, durability, and flexibility. The PA family includes several iterations including PA6, PA6,6, PA6,10, PA10,10, etc., with PA6 and PA6,6 being the most common.^1^ These polymers are used throughout the automotive, aerospace, telecommunications, and textile industries.^2^ Despite their many benefits, PAs are highly dangerous when exposed to a flame, as they promote rapid fire propagation due to high heat release rates and molten flaming drops of material (i.e., melt dripping).^3^ For these reasons, the development of effective flame retardant (FR) treatments for PA substrates is a necessity.

The primary flame retardant approaches for PAs are copolymerization with FR monomers,^4,5^ blending with additives,^6,7^ and surface coatings.^8,9^ While copolymerization and blending can be effective in preventing flame spread by integrating FR components into the bulk material, they often alter nylon’s favorable mechanical properties or are less industrially compatible.^4^ Coatings localize FR protection at the material’s surface where combustion occurs, preserving the performance of the underlying material. For these reasons, the present study is focused on the development of a coating that can be applied to a wide variety of polyamides.

One class of effective flame-retardant surface treatments is intumescent systems, which form a protective and insulating char layer when exposed to flame. Intumescents typically consist of an acid source, a carbon source, and a blowing agent. During combustion, the acid source catalyzes cross-linking and dehydration of the hydroxyl-rich carbon source to generate char, which is subsequently expanded by noncombustible gases released from the blowing agent. The resulting intumescent char acts as a thermal barrier, reducing heat and mass transfer to the underlying substrate.^10^ Depositing intumescent coatings via a water-based polyelectrolyte complex (PECs) was initially demonstrated on cotton more than a decade ago.^11^ Two polymers of opposite charge are combined at a pH where they do not strongly associate, forming a stable PEC solution. A substrate can be dip coated into this mixture before being “cured” in a second solution held at a pH that induces the ionic interaction between the two polymers and forms a conformal coating on the substrate’s surface.^12^

Tannic acid (TA) is a polyphenol derived from the tannins of plants that has shown promise as a bioderived flame retardant additive due to its excellent char-forming ability.^13^ Chitosan (CS) is a biopolymer obtained through the deacetylation of chitin harvested from marine crustaceans.^14^ Both TA and CS have become popular bioderived flame retardant components.^13,15^ While neither material alone provides significant flame retardancy to synthetic textiles, their combination with a phosphate source can promote the formation of an intumescent char layer during combustion.^16^ Guanidine phosphate (GP), a bioderived phosphorus-containing compound, serves as an effective acid source and has previously demonstrated flame-retardant efficacy in bioderived coatings for nylon-cotton blend fabrics.^17^ This study outlines a highly effective intumescent flame retardant for PA6,6 textiles that can be deposited in a simple two-step dip-coating PEC process. All components used are derived from biological feedstocks, eliminating the need for petroleum derived flame-retardant materials.

## 2 Experimental Methods

### 2.1 Materials

Spun polyamide 6,6 (PA 6,6) woven fabric (plain weave) was purchased from TestFabrics, Inc. (West Pittston, PA). Tannic acid [TA] (ACS Reagent, 98%) was purchased from Sigma-Aldrich (Milwaukee, WI), while guanidine phosphate [GP] (98%) was purchased from Avantor VWR (Radnor, PA). Chitosan [CS] (95% deacetylated) was purchased from Greentech Biochemicals (Qingdao, China). All solutions were prepared using 18 MΩ deionized [DI] water and pH adjustments were performed by adding 5 M HCl dropwise. TA solutions were adjusted to pH 2. CS solutions were prepared by first dissolving CS powder in a 3.6% acetic acid solution before adjusting the pH of the solution to pH 2. GP solutions were adjusted to pH 4. TA and CS solutions were combined and stirred to form a stable transparent PEC solution. GP solutions were kept separate to be used as a second dip coating/ionic cure step. Solutions of CS, TA, and GP were prepared to achieve weight ratios of 1:5:7, 1:9:7, 1:7:7, 1:7:5, and 1:7:9 CS:TA:GP at 15% solids. A table outlining the concentrations and amounts of solution used to make 3 samples for VFT is outlined in Table S1.

### 2.2 Coating Deposition

Prior to coating deposition, PA6,6 fabric was rinsed with DI water and dried in a 70 °C oven for ∼30 min (or until completely dry). The fabric was placed in the TA-CS PEC solution and allowed to soak for 5 min. The liquor ratio for all solutions was 1:20, corresponding to 20 mL of solution for every gram of fabric. After the sample was removed from the PEC solution, it was dried at 70 °C. Finally, the sample was dipped in the GP solution for 5 min, dried, rinsed with DI water, and dried again. This process is summarized in Figure 1.

**Figure 1:**
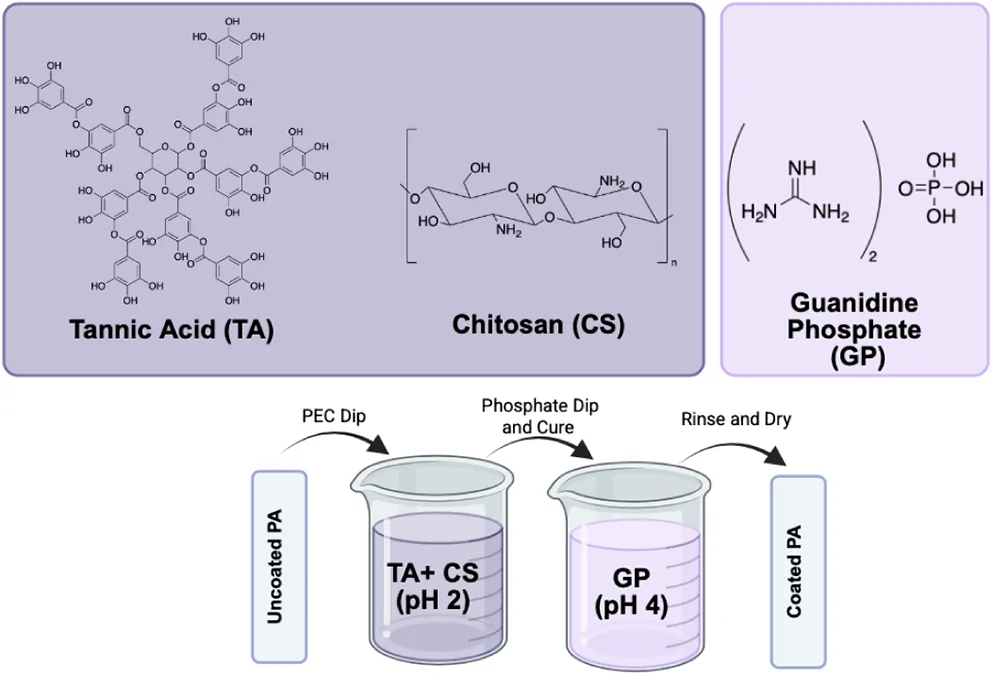
Chemical structures of coating materials and schematic of coating procedure.

### 2.3 Thermal Characterization

Vertical flame testing (VFT) was used to evaluate the flammability of PA6,6 samples (30.5 × 8.9 cm) according to ASTM D6413-15. Limiting Oxygen Index (LOI) testing was performed using an FTT oxygen index apparatus (Fire Testing Technology, E Grinstead, UK), following the guidance of ASTM D2863. All samples were subjected to VFT and LOI testing, but only the samples with the most promising flame performance were characterized further. Thermal degradation and gas evolution of samples was evaluated using a TA Instruments TGA 5500 with a Discovery mass spectrometer (TA Instruments, New Castle, DE) under an oxidative environment. Thermogravimetric analysis (TGA) samples (∼5 mg) were subjected to an isotherm at 100 °C for 10 min to remove any residue water before ramping 10 °C/min to 600 °C. Microscale combustion calorimetry (MCC) was used to assess the material’s flammability according to Method A of ASTM D7309, using a MCC-3 (Deatak, McHenry, IL). Cone calorimetry (CC) experiments were conducted in the Deatak CC-2 Cone Calorimeter at The University of Dayton Research Institute (Dayton, OH) at a 35 kW·m^–2^ heat flux according to ASTM E1354-25.

### 2.4 Other Characterization

Scanning electron microscopy (SEM) was performed to analyze the coating’s morphology and the structure of the resulting postburn char using a JEOL JSM-7500F field emission SEM (JEOL, Tokyo, Japan). Prior to imaging, samples were sputter coated with 5 nm of a Pt–Pd alloy. Elemental analysis of N and P content was conducted with electron dispersive spectroscopy (EDS). Samples were further imaged using a JEOL 1200 EX TEM (JEOL, Tokyo, Japan) transmission electron microscope (TEM). TEM samples were prepared by staining a coated PA6,6 fiber with uranyl acetate, then embedding the sample in Epofix resin (EMS, Hatfield, PA, USA) before sectioning the resin with a Leica UC7 ultramicrotome (Leica, Wetzlar, Germany). Strength, elongation, and stiffness of fabric samples were evaluated with an Instron 6800 Universal Testing Machine (Instron, Norwood, MA), using a 2 kN load cell. The tensile test samples (12 × 2 cm) were tested according to ASTM D5034 with a testing speed of 5 mm/min. All characterization was performed in triplicate.

## 3 Results and Discussion

### 3.1 Coating Deposition

There is an average PEC add-on weight ranging from 20 to 31.5% depending on the ratio of FR components (Table 1). Increasing the TA content from a CS:TA:GP ratio of 1:5:7 to 1:9:7 increases the add-on from 26.1 to 29.1 wt %. Likewise, increasing the GP content to a 1:7:9 ratio results in the highest add-on weight (31.5 wt %), whereas reducing the GP content to a 1:7:5 ratio yields the lowest add-on (20.0 wt %). SEM images of uncoated PA6,6 reveal the textile weave with some disorganized fibers (Figure S1). In lower magnification images, the coated PA6,6 samples appear similar to uncoated PA6,6, but at higher magnifications, coated samples show aggregates of coating along the fibers and some fiber bridging spanning gaps between fibers. Cross-sectional TEM images display a conformal coating surrounding individual nylon fibers, confirming successful deposition and incorporation of GP into the coating, as the uranyl acetate stain used selectively binds to phosphate species (Figure S1).^18,19^ Successful GP incorporation is further supported by elemental mapping, which shows the presence of phosphorus only in coated PA6,6 samples (Figure S2). Prior to coating, there is not a detectable phosphorus signal, however upon coating deposition, an average of 0.37% of the sample’s atomic makeup is phosphorus.

**Table 1:** Summary of Flame Testing Results

Weight Ratio (CS:TA:GP)	Weight Gain (%)	Char Length (in)	Afterburn (s)	Char Residue (%)	Melt Dripping?	LOI (%)
Uncoated (0:0:0)	0.0 ± 0.0	5.2 ± 0.1	15.1 ± 1.7	76.2 ± 2.1	Yes	23.5 ± 0.1
1:5:7	26.1 ± 1.7	5.0 ± 0.8	0.0 ± 0.0	96.7 ± 1.5	No	26.3 ± 0.1
1:9:7	29.1 ± 1.5	4.8 ± 1.2	0.0 ± 0.0	96.4 ± 1.2	No	27.0 ± 0.1
1:7:7	25.1 ± 1.8	6.2 ± 5.2	8.7 ± 15.0	87.8 ± 17.9	No	27.1 ± 0.1
1:7:5	20.0 ± 0.4	12.0 ± 0.0	21.7 ± 1.2	62.3 ± 7.0	No	25.6 ± 0.1
1:7:9	31.5 ± 2.1	6.5 ± 3.8	6.7 ± 6.5	80.7 ± 9.8	No	25.9 ± 0.1

Given the p*K*_a_’s of TA and CS (6 and 6.5, respectively),^20,21^ TA is largely protonated and therefore uncharged at pH 4, making it unable to participate in ionic interactions. Consequently, PEC coating formation is likely driven primarily by hydrogen bonding between the polymers and guanidine phosphate rather than traditional electrostatic interactions.

To evaluate the coating’s influence on the mechanical properties of PA6,6, tensile testing experiments were performed on coated and uncoated substrates. The strength, elongation, and stiffness are summarized in Figure 2. Overall, tensile strength, stiffness, and sample elongation increase for coated samples. The increase in tensile strength and stiffness suggest that the coating promotes load transfer between adjacent fibers. This behavior is consistent with a fiber bridging mechanism, where the coating spans gaps between adjacent fibers and limits fiber slippage.^22^ The reduced slippage contributes to the decreased variability observed for coated samples during tensile testing (Figure 2b). The increase in elongation also implies that the coating is not simply making the textile more brittle, but rather improving stress distribution between fibers, allowing greater deformation before fiber failure. Similar fiber bridging effects have been reported for other textile systems.^23,24^

**Figure 2:**
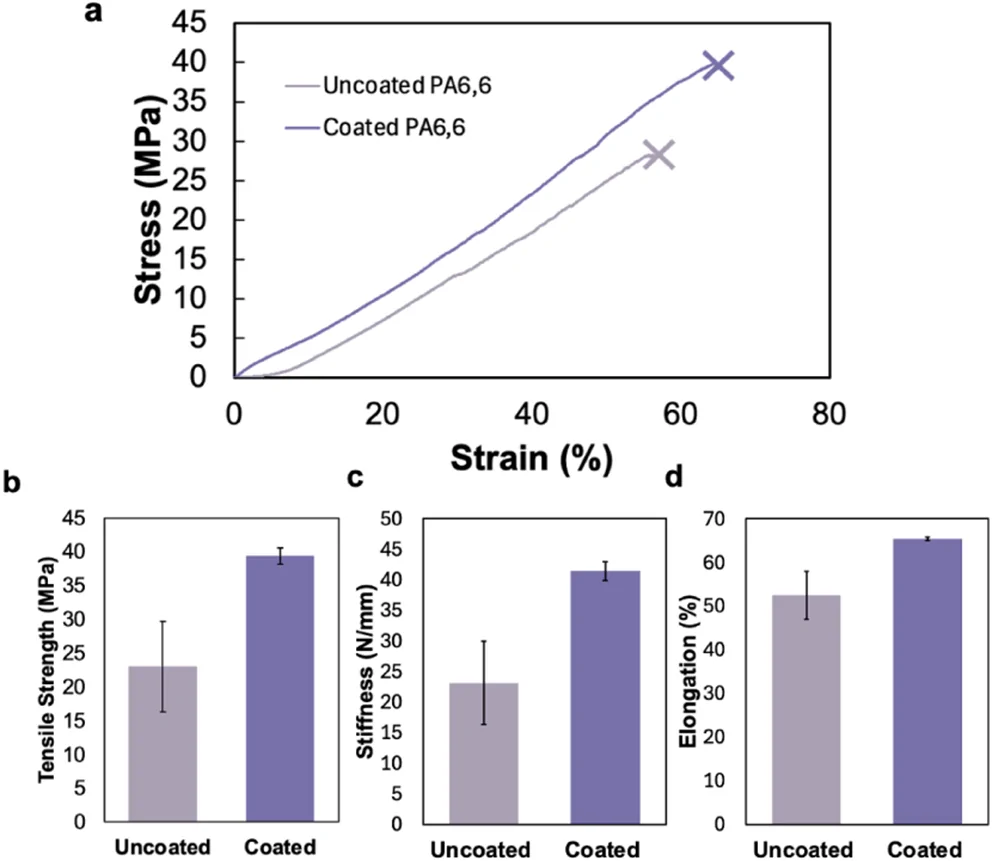
(a) Representative stress–strain curves for coated and uncoated PA6,6 samples. Sample failure is marked with an “X”. (b) Average maximum tensile strength, (c) average stiffness, and (d) average sample elongation.

Although increased stiffness can be a limitation of a textile system, as the “hand” or feel of the fabric is important to its utility. Manual agitation and conditioning of PEC-treated textiles has been shown to largely restore the stiffness of a PEC-coated substrate to that of the uncoated substrate’s stiffness, indicating that although the coating alters the fabric’s hand, these effects can be mitigated through post-treatment conditioning.^25,26^

### 3.2 Flammability and Thermal Stability

The flame performance of various ratios of CS:TA:GP were assessed to determine which ratio yields the best flame performance. During vertical flame testing (VFT), samples were exposed to a direct flame for 12 s and the resulting char length, afterburn, and occurrences of melt dripping were recorded. Upon flame exposure, uncoated PA6,6 ignites and melts away from the flame source, releasing molten flaming droplets that are expected to propagate a flame to any nearby materials in a full-scale fire setting. Samples treated solely with the CS/TA solution display similar behavior, melting away from the flame and releasing molten droplets. In contrast, GP alone does not exhibit melt dripping, but allows the flame to travel up the majority of the sample, resulting in low char yields (Figure S3, Table S2). These control experiments demonstrate that neither CS/TA or GP alone provide sufficient flame retardancy to prevent flame propagation or melt dripping. All tested ratios of combined CS, TA, and GP eliminate melt dripping and increased the char yields, indicating a synergistic interaction between the three components. CS and TA provide carbon-rich char precursors, while GP is both phosphorus and nitrogen rich, promoting intumescent char formation as both an acid source and blowing agent. The phosphoric acid of GP can catalyze the dehydration and carbonization of CS and TA, promoting the rapid formation of a thermally stable protective char residue. This char layer prevents melt flow by forming a physical barrier before significant PA6,6 melting can occur. Evidence of this can be seen in the SEM images discussed in Section 3.3.

As shown in Figure 3 and Table 1, only the 1:5:7 and 1:9:7 ratios are consistently self-extinguishing and have similar char residues (96.7 and 96.4, respectively). The average char length is shorter for the 1:9:7 samples, which has the highest tannic acid content. These findings are supported by the LOI measurements, where the 1:9:7 sample exhibits the highest LOI value of 27.0, representing a 3.5 percentage point increase from uncoated PA6,6. The 1:7:7 ratio samples has an equally high LOI value but with inconsistent VFT performance resulting in a larger average char length (6.2 in.) than the 1:9:7 samples (4.8 in.). For this reason, the samples with the 1:9:7 CS:TA:GP ratio were chosen for further analysis.

**Figure 3:**
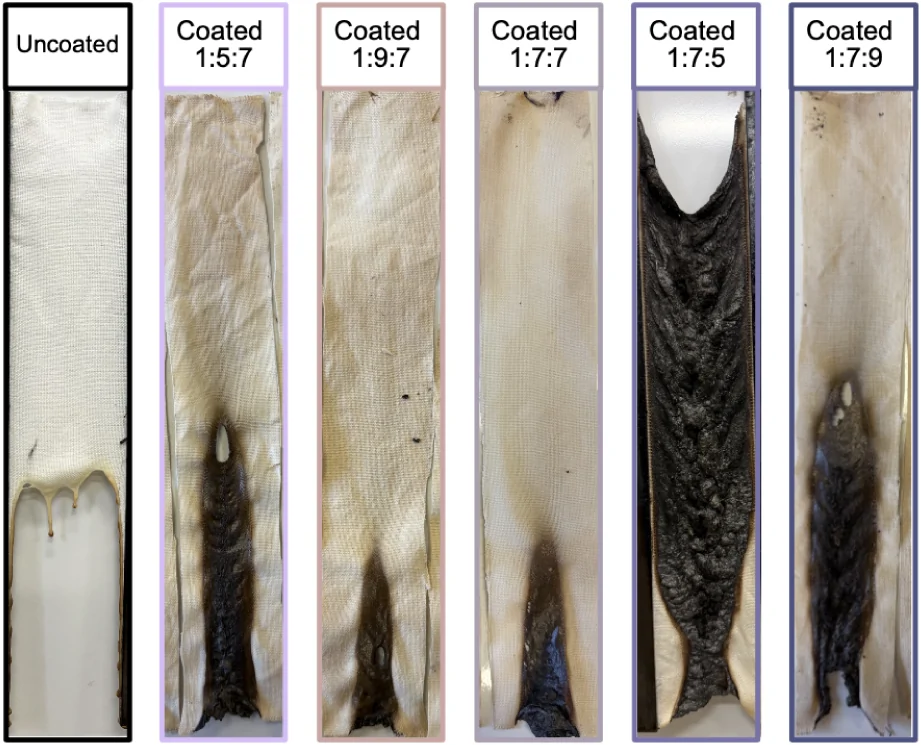
Representative digital images of vertical flame testing results. Ratios represent the weight ratios of CS:TA:GP.

The efficacy of this PEC coating in decreasing flammability was further quantified using micro scale combustion calorimetry (MCC). A single heat release event is observed for uncoated PA6,6 at 484 °C, with a peak heat release rate (pkHRR) of 628.3 W·g^–1^ (Figure 4, Table 2). This rapid rate of heat release reflects the fast combustion of PA6,6 and its inherent flammability.^27^ Coated PA6,6 shows two heat release events, one at 388.5 °C and a second at 468 °C. The maximum pkHRR of coated PA6,6 is 286.6 W·g^–1^, approximately 2.9x less than that of uncoated PA6,6. Furthermore, the total heat released (THR) and fire growth capacity (FGC) are decreased by 42% and 58%, respectively. The lower temperature onset of heat release for coated PA6,6 is characteristic of an intumescent system, as the phosphates of GP promote char formation at a lower temperature than PA6,6 combustion begins.^17,28^ Intumescence is further evidenced by an increase in the char yield, from an average of 3.6% to 15.2% from uncoated to coated PA6,6. Increased char formation suppresses the evolution of flammable volatile decomposition products, thereby decreasing the amount of fuel available for combustion, contributing to reduced heat release.

**Figure 4:**
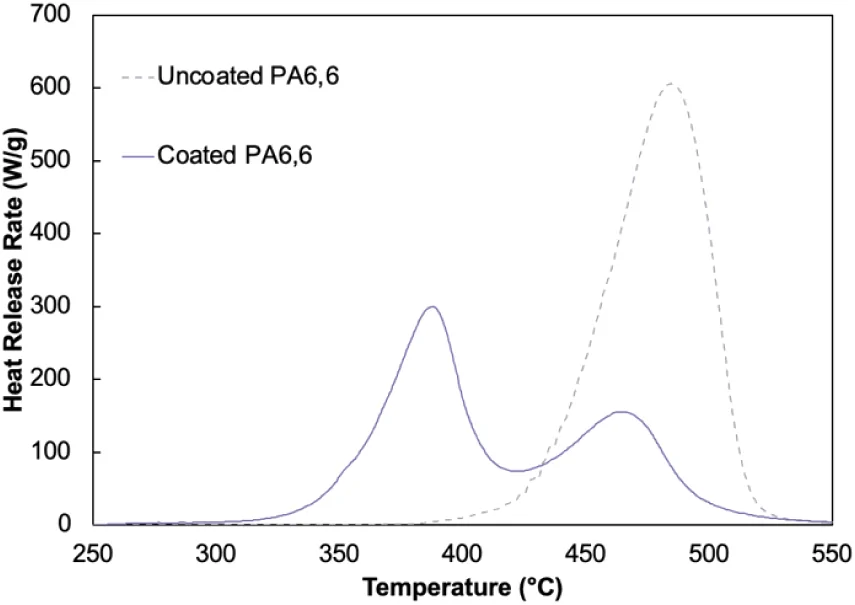
Representative heat release rate as a function of temperature for uncoated and coated PA6,6.

**Table 2:** Summary of Micro Combustion Calorimetry Results

	pkHRR (W·g^–1^)	THR (kJ·g^–1^)	FGC (J·g^–1^·K^–1^)	T_pkHRR_ (°C)	Char Yield (%)
Uncoated PA6,6	628.3 ± 32.9	30.1 ± 1.3	516.9 ± 10.7	484.2 ± 6.1	3.6 ± 1.0
Coated PA6,6	286.6 ± 29.8	17.6 ± 6.4	215.4 ± 9.1	388.5 ± 1.6	15.2 ± 6.0

While MCC provides valuable information about milligram-scale samples under controlled pyrolysis conditions, cone calorimetry (CC) evaluates the burning behavior of larger specimens exposed to an external heat flux, providing more representative assessment of real fire performance. For this reason, CC testing was performed at a heat flux of 35 kW·m^–2^, which is typically found during the early stages of a developing fire.^29^ Figure 5 and Table 3 summarize this full size cone data. Uncoated PA6,6 ignites within 60.7 s and exhibits a sharp HRR peak of 261 kW·m^–2^. In contrast, the coated PA6,6 displays a substantially reduced and broadened HRR profile, with a pkHRR of 114 kW·m^–2^, a 56% decrease in the rate of heat release. The time to ignition (TTI) for coated PA6,6 is significantly accelerated compared to uncoated PA6,6, further supporting the assertion that this coating behaves in an intumescent manner. Despite improvements in pkHRR and THR, the coating causes a substantial increase in total smoke release (TSR), rising from 43 to 119 m^2^·m^–2^. Increased smoke production is sometimes observed in systems that promote condensed-phase char formation, since incomplete combustion can generate larger quantities of smoke and carbonaceous particles.^28,30^ This is a limitation of the system that could be addressed through the addition of smoke suppressant additives such as zinc stannate.^31^

**Figure 5:**
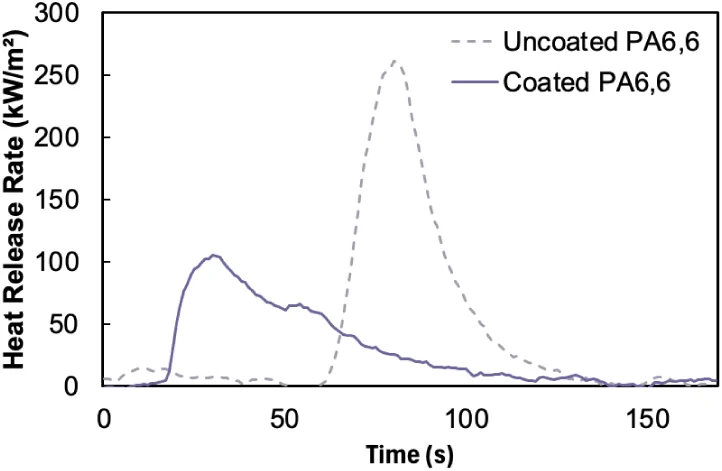
Representative heat release rate as a function of time, as determined by cone calorimetry.

**Table 3:** Summary of Cone Calorimeter Data

	Time to Ignition (s)	pkHRR (kW·m^–2^)	THR (MJ·m^–2^)	Total Smoke Release (m^2^·m^–2^)
Uncoated PA6,6	60.7 ± 4.5	261.0 ± 2.0	7.0 ± 0.1	43 ± 4.2
Coated PA6,6	21.8 ± 3.4	114.0 ± 15.0	5.4 ± 0.6	119 ± 5.1

Thermal degradation and decomposition byproducts of coated and uncoated PA6,6 were evaluated in an oxidative environment via thermogravimetric analysis-mass spectrometry (TGA-MS) (Figure 6). Mass spectrometry ion currents for 18 *m*/*z* and 44 *m*/*z* were plotted as a function of temperature (Figure 6b, c). These masses are understood to primarily represent H_2_O and CO_2_, respectively.^32^ Uncoated PA6,6 has two major degradation events. The first has an onset around 300 °C, followed by a shoulder before a major thermal degradation event around 400 °C, which can be attributed to chain scission reactions along the polymer backbone from breakage of C–N linkages.^1,33^ Figure 6b supports this conclusion, as an increase in ion current is observed at 300 °C and spikes again around 400 °C. The loss of water at these temperatures indicates dehydration reactions and char formation. Additionally, the major mass loss event was accompanied by increased ion currents of 55 and 82 amu. These masses are understood to correspond to fragments of polyamide chains, further evidencing C–N bond breakage.^33^ At 500 °C, there is another mass loss event corresponding to an increase in CO_2_ detection (Figure 6c), suggesting substrate volatilization and oxidative degradation of char.^34^ Coated PA6,6’s first major thermal degradation event occurs at a temperature approximately 120 °C lower than that of uncoated PA6,6. This accelerated degradation is characteristic of intumescent systems, as char formation is catalyzed by the phosphates of GP. The major degradation event is followed by a stepwise degradation, with mass loss around 400 and 500 °C, leaving behind 22.4 wt % residue. The uncoated PA6,6 left behind only 3.4%, indicating the coating’s ability to promote formation of a robust protective char that resists oxidative degradation. For coated PA6,6, the initial release of H_2_O and CO_2_ at low temperatures is greatly reduced, indicating that char formation reactions are being favored over gas-evolving decomposition.^28^ Around 400 °C, an increase in the ion current associated with H_2_O is observed, coinciding with a degradation event likely from the polymer backbones in the coating and substrate thermally decomposing. At this temperature, there is also a spike in the ion current associated with CO_2_, suggesting TA decarboxylation and some substrate volatilization at high temperatures.^35^ Overall, TGA-MS demonstrates that the TA/CS/GP coating promotes intumescent char formation, suppresses volatile decomposition products, and significantly increases char yield compared to uncoated PA6,6.

**Figure 6:**
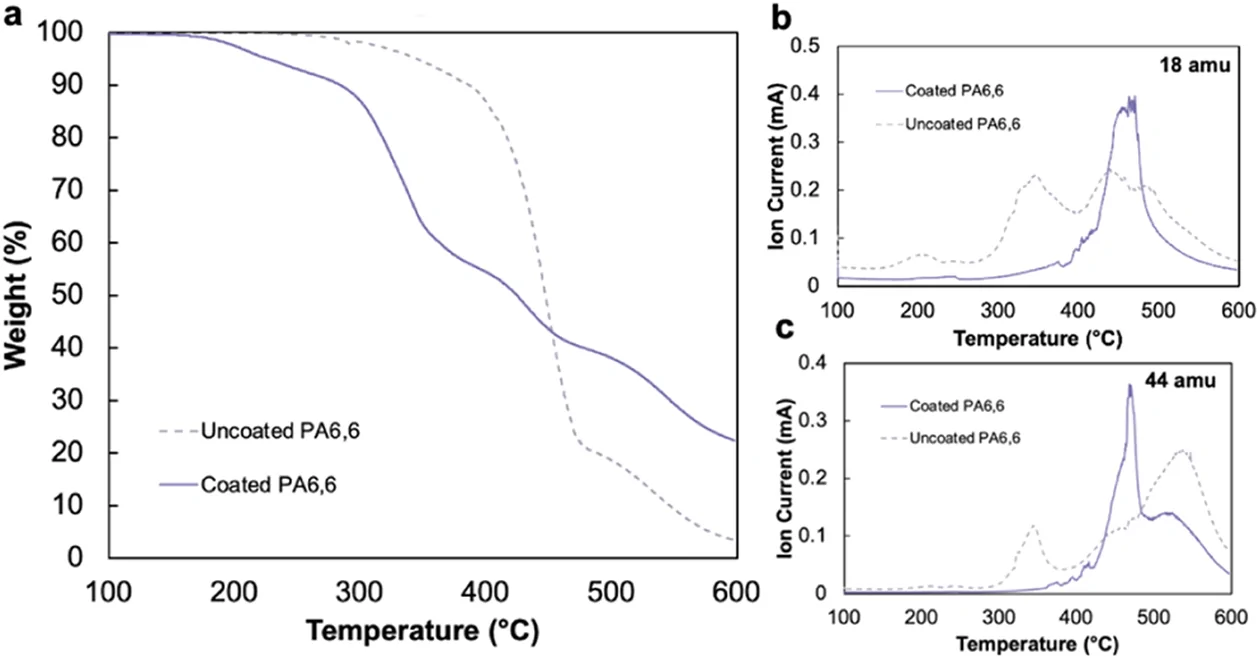
(a) TGA thermograms of uncoated and coated PA6,6 under an air atmosphere. (b,c) Mass normalized ion current collected by TGA-MS at 18 and 44 amu.

### 3.3 Flame Retardant Mechanism

SEM images were obtained for uncoated and coated PA6,6 in the regions indicated in Figure 7. With uncoated PA6,6, evidence of melting is apparent, as the textile weave is no longer distinguishable due to fiber fusion in regions exposed to the flame. Similar melting is observed along the perimeter of the char region in coated samples. In the area directly exposed to the flame, there is a fluffy, contiguous char that masks the fabric weave. Images of this char, display evidence of bubbling which is indicative of blowing agent activation during intumescence.^36^

**Figure 7:**
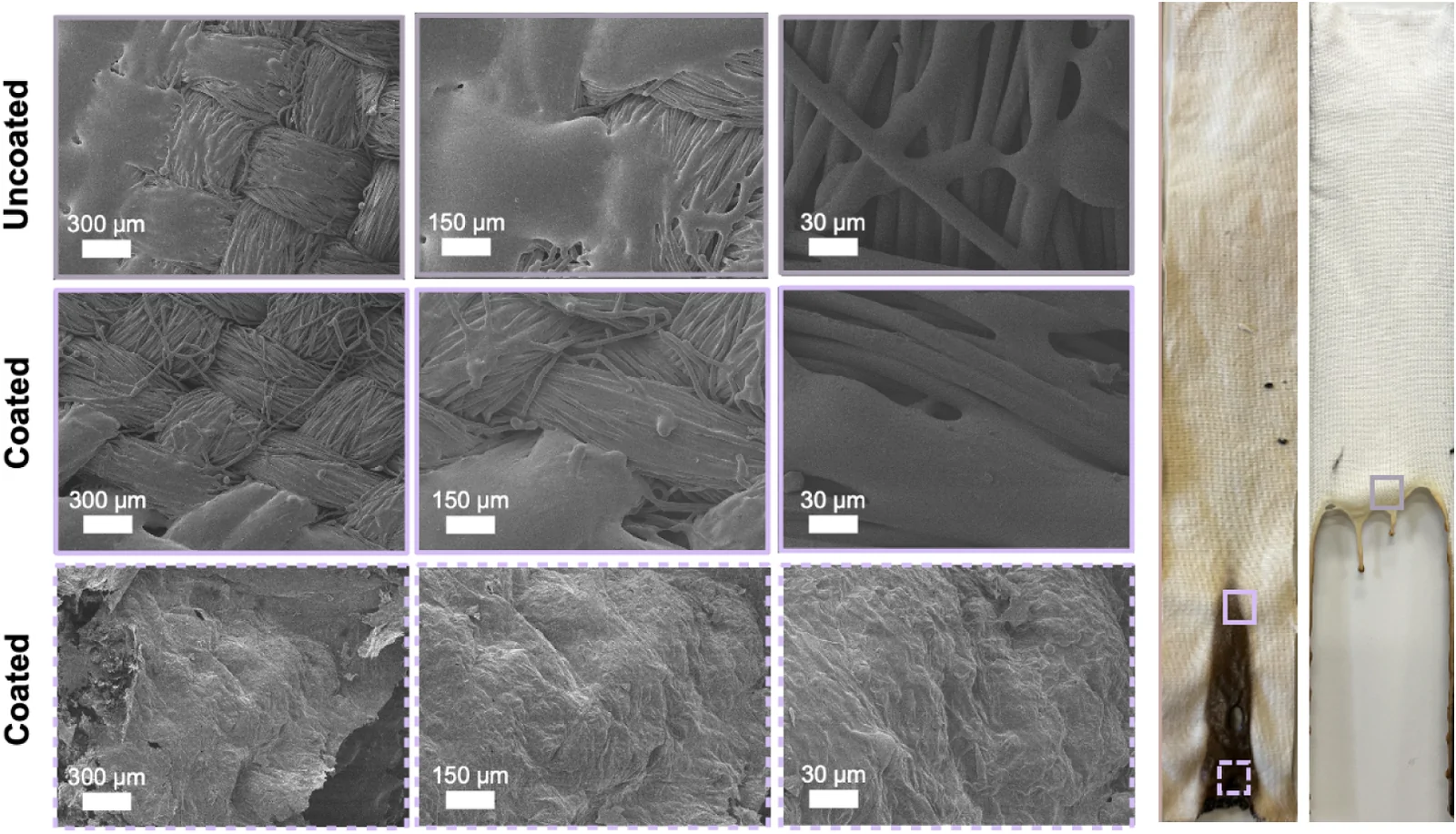
SEM images of uncoated and coated PA6,6 with boxes demarking where samples for coated PA6,6 were obtained.

The coating’s char-promoting ability is further demonstrated following cone calorimeter testing. Uncoated PA6,6 is almost entirely volatilized following testing, however, coated samples display impressive char formation and the generation of macroscopic char bubbles (Figure S4). This expanded char structure corresponds with a substantial reduction in pkHRR observed during CC, suggesting that the coating effectively suppresses heat and mass transfer during burning.

Elemental analysis provides additional support for an intumescent mechanism. Following VFT, the phosphorus to nitrogen ratio increases more than 3-fold relative to the unburned coating (Table S3), suggesting preferential nitrogen loss during thermal decomposition and blowing-agent activation. Collectively, these findings support a flame retardant mechanism in which GP catalyzes char formation while generating blowing gases that expand a tannic acid and chitosan-derived protective char layer, reducing substrate volatilization, heat release, and flame propagation.

## 4 Conclusions

An entirely bioderived intumescent flame-retardant coating composed of tannic acid, chitosan, and guanidine phosphate was successfully deposited onto PA6,6 textiles through a simple two-step dip-coating process. The coating conformally coats individual fibers. Among the compositions investigated, the 1:9:7 CS:TA:GP weight ratio formulation exhibits the most consistent flame-retardant behavior, eliminating melt dripping, achieving self-extinguishing performance during vertical flame testing, and increasing the LOI to 27.0% (from the original 23.5%). MCC and cone calorimetry reveal major reductions in heat release and fire growth behavior, while TGA-MS reveals accelerated char-forming reactions and suppression of volatile decomposition products. The coating promotes the formation of a thermally stable intumescent char. Although smoke production increased, the coating suppresses rapid flame propagation and prevents molten dripping, two critical fire hazards associated with PA textiles. Future work is required to reduce smoke production and improve coating durability during laundering, which will be studies unto themselves. Overall, this study’s findings demonstrate that bioderived intumescent coatings can provide an environmentally friendly alternative to conventional flame-retardant treatments for synthetic textiles. The TA/CS/GP system described herein offers a scalable and versatile strategy for improving the fire safety of polyamides, while minimizing dependence on petroleum-derived chemistries.

## Supplementary Material



## Notes

Coating formulation table (Table S1); SEM and TEM images of coating deposition (Figure S1), EDS mapping (Figure S2), images of controls after VFT (Figure S3), summary of VFT data (Table S2), images of samples post cone calorimetry testing (Figure S4), and relative elemental concentrations (Table S3) (PDF)
