# Supplementary material for: Entirely Bioderived Intumescent Flame-Retardant Coating for Polyamide Textiles
Source: ACS Polym Au. 2026 Aug 26;6(5):1499–505. doi: 10.1021/acspolymersau.6c00154 (PMC13673877; doi:10.1021/acspolymersau.6c00154)
Supplement: Supplementary file 1 [file lg6c00154_si_001.pdf]

Entirely Bioderived Intumescent Flame-Retardant Coating for Polyamide Textiles

Margaret J. Karim[a], Sarah Fisher[a], Kristin L. Smith[b], Dallin L. Smith[a] and Jaime C. Grunlan[acd]*

[a] Department of Chemistry, Texas A&M University, College Station, TX, USA 77843

[b] Department of Biochemistry and Biophysics, Texas A&M University, College Station, TX, USA 77843

[c] Department of Materials Science and Engineering, Texas A&M University, College Station, TX,USA 77843

[d] Department of Mechanical Engineering, Texas A&M University, College Station, TX, USA 77843

[*] Corresponding author, jgrunlan@tamu.edu

Table S1. Concentrations and amounts of solutions needed for vertical flame test samples.

Target Ratio in Final Coating (CS:TA:GP)	Amount of CS Solution (g)	Concentration of CS Solution (%)	Amount of TA Solution (g)	Concentration of TA solution (%)	Amount of GP Solution (g)	Concentration GP Solution (%)
1:5:7	150	2.3	150	11.5	300	8.0
1:9:7	150	1.8	150	15.9	300	6.2
1:7:7	150	2.0	150	14.0	300	7.0
1:7:5	150	2.3	150	16.1	300	5.8
1:7:9	150	1.8	150	12.4	300	7.9

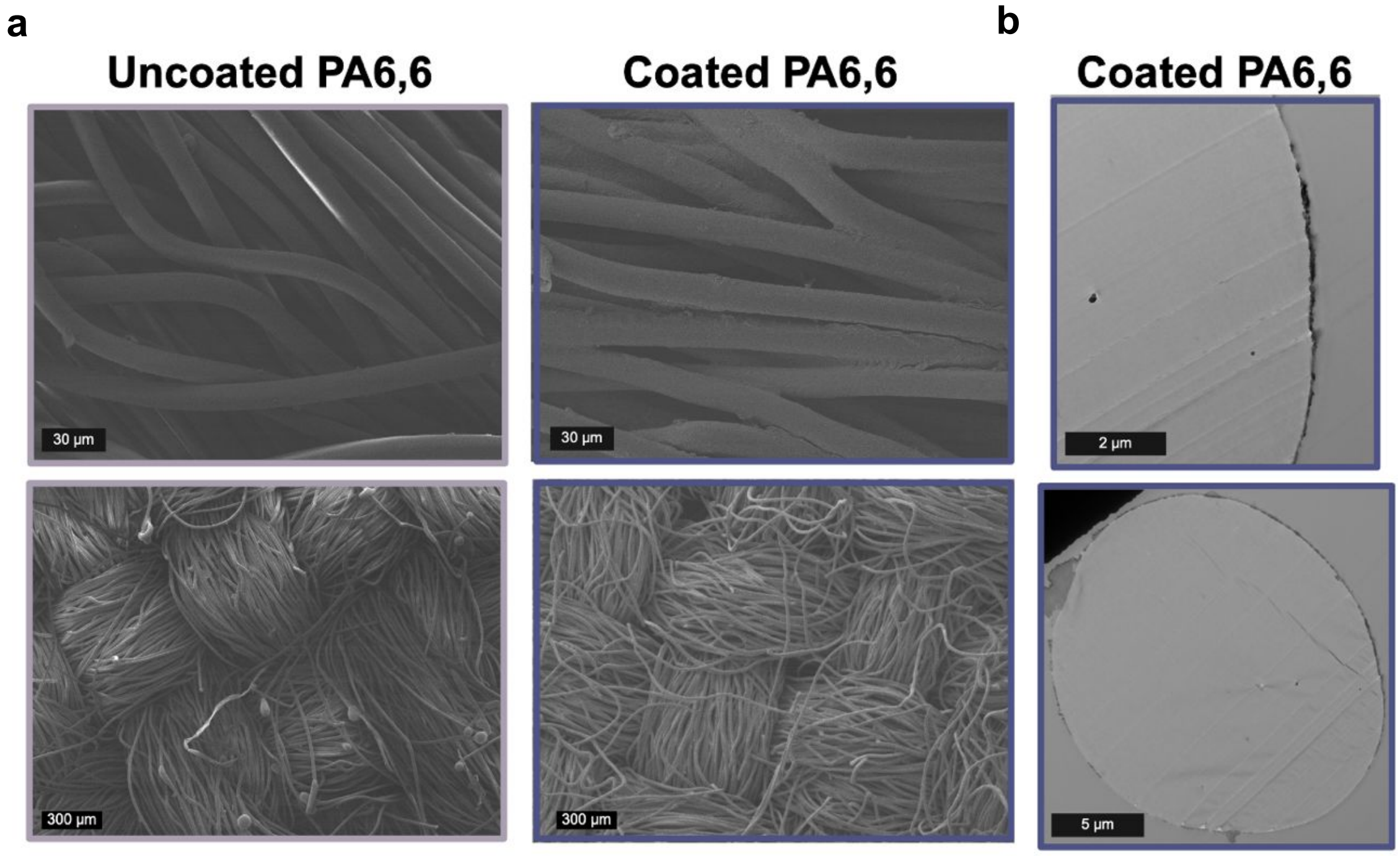

Figure S1. a) SEM images of uncoated and coated PA6,6 textiles. B) cross sectional TEM images of a single coated PA6,6 fiber that has been treated with uranyl acetate stain and imbedded in epoxy at 10,000X (top) and 3,000X (bottom) magnification.

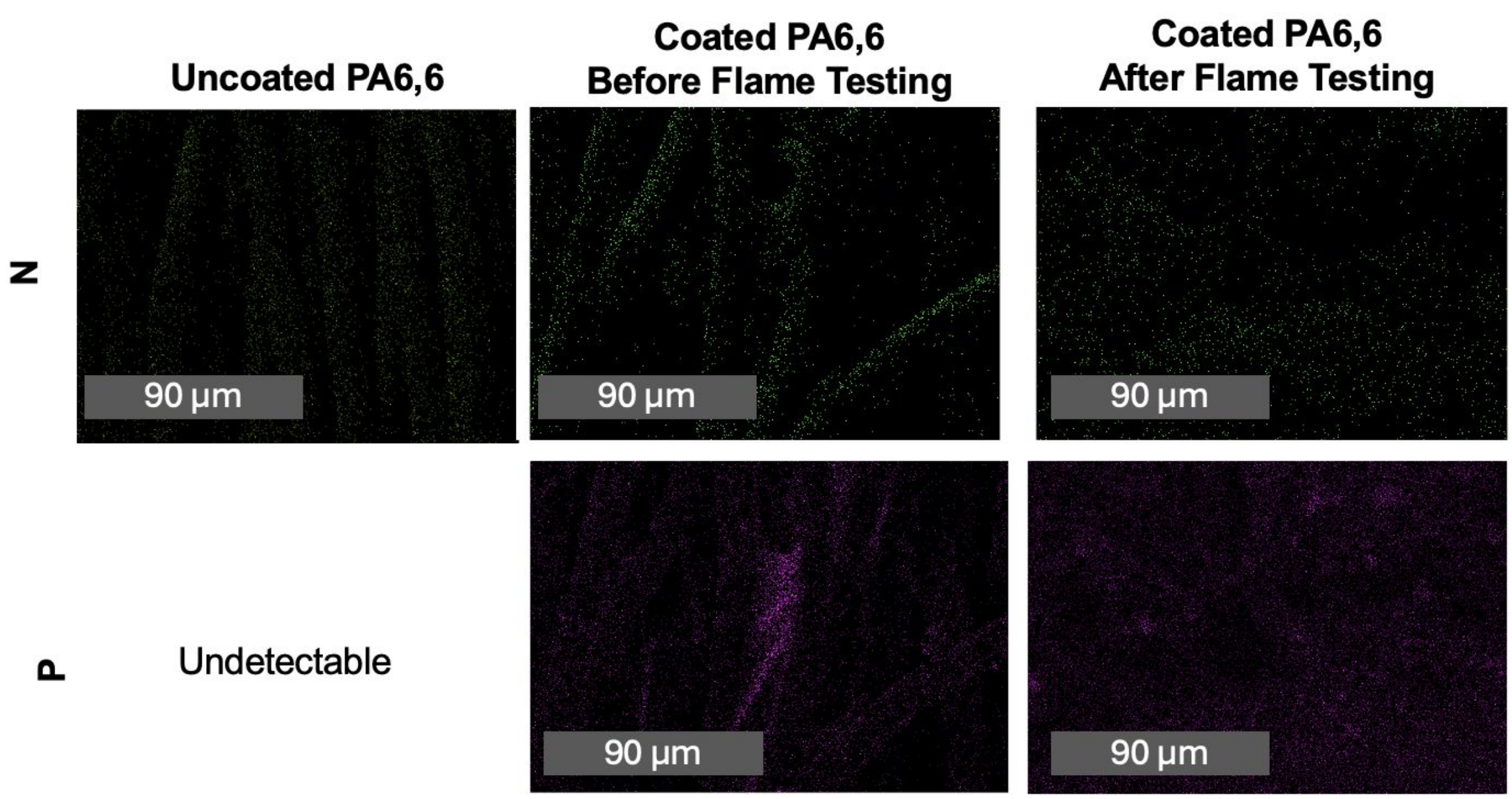

Figure S2. Elemental mapping of nitrogen and phosphorus, before and after flame testing, for coated PA6,6 samples.

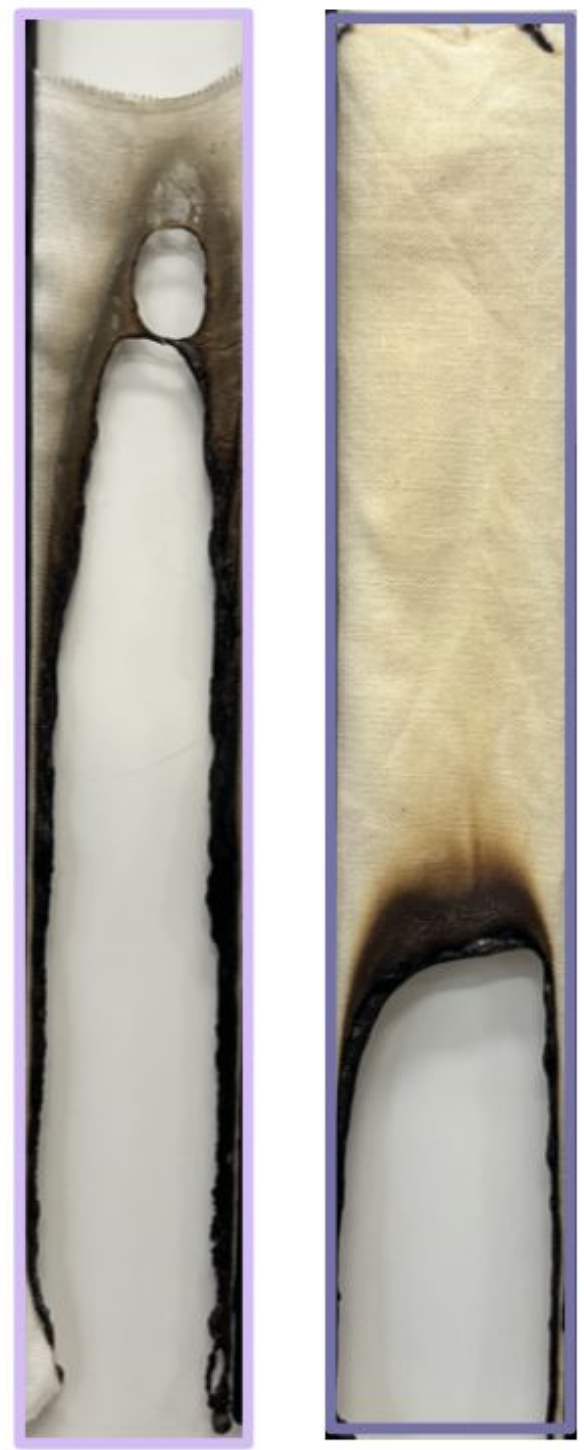

Figure S3. Representative digital images of control vertical flame testing results.

Table S2. Summary of control sample vertical flame testing results.

	**Weight gain (%)**	**Char Length (in)**	**Afterburn (s)**	**Char residue (%)**	**Melt dripping?**
Only GP	17.2 ± 0.9	5.3 ± 0.4	25.3 ± 1.2	67.7 ± 0.3	No
Only CS/TA	28.4 ± 1.7	5.3 ± 0.4	30.0 ± 2.0	68.1 ± 14.6	Yes

a b

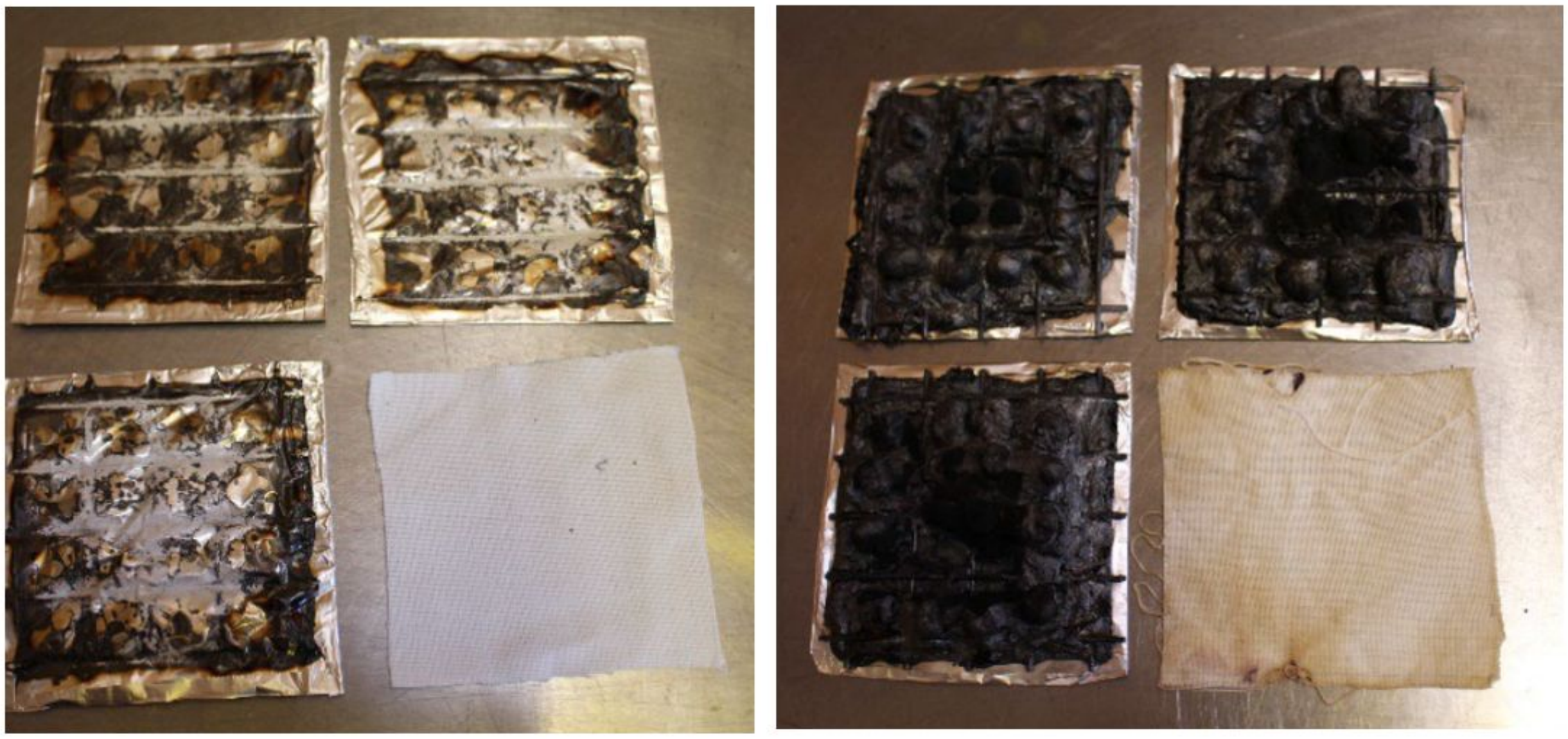

Figure S4. a) Images of uncoated PA6,6 post cone testing with sample in bottom right quadrant representing sample before testing. b) Images of coated PA6,6 post cone testing with sample in bottom right quadrant representing sample before testing.

Table S3. Relative phosphorus and nitrogen concentrations as determined by EDS.

Sample	Relative Phosphorus Concentration (%)	Relative Nitrogen Concentration (%)	P:N Ratio
Uncoated PA6,6 Before Flame Testing	Undetectable	14.3 ± 0.6	NA
Coated PA6,6 Before Flame Testing	0.35 ± 0.07	14.6 ± 0.7	0.02
Coated PA6,6 After Flame Testing	0.87 ± 0.07	12.2 ± 0.5	0.07
